# Comparison of Efficacy and Safety of Two Different Enoxaparin Products in Prevention of Venous Thromboembolism Following Major Obstetric-gynecological Surgeries: An Open-label Randomized Clinical Trial

**DOI:** 10.22037/ijpr.2019.111902.13417

**Published:** 2019

**Authors:** Manoochehr Abdolvand, Ashraf Aleyasin, Mohammad Reza Javadi, Mohammad Solduzian, Seyed Hossein Hosseini, Zohreh Ziaei, Samira Chaibakhsh, Kheirollah Gholami

**Affiliations:** a *Department of Clinical Pharmacy, School of Pharmacy, Tehran University of Medical Sciences, Tehran, Iran. *; b *Department of Obstetrics and Gynecology, Dr. Shariati Hospital, Tehran University of Medical Sciences, Tehran, Iran. *; c *Research Center for Rational Use of Drugs, Tehran University of Medical Sciences, Tehran, Iran. *; d *Neuromusculoskeletal Research Center, Iran University of Medical Sciences, Tehran, Iran.*

**Keywords:** Venous thromboembolism, Enoxaparin, Primary Prevention, Surgery, Cesarean Section

## Abstract

Venous thromboembolism (VTE) occurs in about 5 percent of patients undergoing major abdominal surgeries. Prophylaxis of VTE is recommended using unfractionated heparin (UF) or low molecular weight heparin (LMWH) in high-risk patients. In spite of advantages and confirmed cost-effectiveness of LMWH, high costs of enoxaparin branded preparations limit its use. We aimed to compare the efficacy and safety of two enoxaparin preparations. In this open-label randomized clinical trial, 200 patients were recruited to recieve PDxane® or Clexane®, 40 mg subcutaneously daily, from the day of surgery for 10 days. The patients were evaluated for VTE occurrence and side effects considering clinical and laboratory examinations at the beginning and day 10. No cases of proximal or distal VTE or life threatening bleeding were observed among 102 and 98 patients who received PDxane® and Clexane®, respectively. The adverse effects observed in PDxane® and Clexane® groups included injection site reactions (rash: *P = *0.97; pain: *P = *0.55 and erythema: *P = *0.33), anemia (*P =* 0.32), hematuria (*P = *0.16), confusion (*P = *0.3), and increased liver transaminases (AST ≥ 3 × ULN: *P = *0.16 and ALT ≥ 3 × ULN: *P = *0.66). In according to the study results PDxane^®^ was of similar efficacy and safety compared to Clexane^®^ in preventing VTE following major obstetric-gynecological surgeries. Considering lower cost of PDxane^®^, it could be a safe and effective alternate for VTE prophylaxis in the patients undergoing such types of surgeries.

## Introduction

Venous thromboembolism (VTE) is a vascular disorder that complicates 20-30 percent of hospital admissions (1). This complication could be presented as either deep vein thrombosis (DVT) or pulmonary embolism (PE) (2). The patients experiencing PE have been shown to have significantly higher rates of mortality in one month following the event and carry this risk for at least 3 years (3). Beside acute complications, the impaired resolution of the thrombus might cause chronic complications such as post-thrombotic syndrome or chronic thromboembolic pulmonary hypertension (4).

The prevalence of DVT has been reported to be about 5% in the patients undergoing major abdominal surgeries (5). Although DVT frequency has been reported to be lower in the patients undergoing less invasive procedures, its incidence was 0.6% percent following abdominal hysterectomy (6). Extended surgery duration is an independent risk factor for the development of DVT (7). The risk of DVT development after cesarean delivery is reported to be 2.6 cases per 1000 operations and is higher compared to normal vaginal delivery (8). 

VTE following surgeries could be prevented using appropriate cost-effective antithrombotic medication. The American College of Obstetricians and Gynecologists (ACOG) guideline recommends VTE prophylaxis pre and post-partum in the patients who have risk factors (such as prolonged immobility, obesity or previous history of VTE) (9). Pharmacologic VTE prophylaxis has been also recommended by the American College of Chest Physicians guidelines in the patients undergoing major gynecologic surgery for either benign or malignant disorders (10).

Both unfractionated heparin (UF) and low molecular weight heparin (LMWH) could be used for VTE prophylaxis. Early reports showed that LMWH was as effective as UFH in preventing VTE in many settings including gynecological surgeries. Moreover, when used at a rational dose, LMWH may not cause more bleeding events compared to UFH (11-15). Efficacy of enoxaparin as a frequently used LMWH has been specifically proven in some surgical settings as well (16).

Counter balancing the advantages of LMWH in the setting of VTE prophylaxis, which includes ease of administration, lack of routine monitoring requirements, improved bioavailability, long half-life, and once daily dosing, it is usually expensive (17). In spite of higher costs, use of Enoxaparin has also been shown to be cost saving compared to UFH in some reports (18, 19). 

Although enoxaparin is now being considered cost effective in prophylaxis of VTE, some of its branded preparations are very expensive and it has also been shown that even generic preparations might not be easily affordable (20).

Therefore, providing less expensive preparations of this medication would be of great help for cost management in health care system considering that ensuranse companies in Iran only cover for the product with the lowest cost (21).

Being similar molecularly and having similar *ex-vivo* effects could not be interpreted as being clinically comparable. Previous comparative studies of a generic preparation with branded enoxaparin, Lovenox®, have shown similar molecular, *in-vitro* and *ex-vivo* characteristics. However, the disparity that was observed in thrombin-activated fibrinolysis inhibitor conversion showed that further research may be required to establish clinical equivalency (22). Hence, efficacy comparisons should be made using clinical trials. In our country different enoxaparin preparations are available that have diverse costs. Whether newly produced preparations could be considered as substitutes for well-known internationally produced brands has been a main concern for our clinicians. Therefore, we aimed to compare the efficacy and safety of a less expensive preparation of enoxaparin, PDxane® that is produced in our country, with Clexane®, which is available in our country by Sanofi Aventis, when used for VTE prophylaxis following gynecologic and obstetric surgeries.

## Experimental

*Study design*

The current study was designed as an open-label randomized clinical trial and carried out in Shariati hospital, Tehran University of Medical Sciences (TUMS), Iran, during the years of 2015 to 2018. TUMS ethics committee reviewed and approved the study protocol and all of the patients gave written informed consents before enrollment in the study. This trial was registered in Iranian Registry of Clinical Trials (IRCT20090914002459N2).

*Study population*

The patients underwent major obstetric-gynecological surgeries (*i.e.* total abdominal hysterectomy, total vaginal hysterectomy, cesarean section, cesarean hysterectomy with or without colporrhaphy) were considered eligible to receive the medication if they were older than 18 years and had risk factors for VTE (prolonged immobility, obesity or previous history of VTE) as it is recommended in ACOG guidelines (9). No scores were used in order to stratify the patients risk category and also inclusion of the patients were done by the ACOG guidelines. 

The patients were excluded from the study if they had history of the previous adverse effects with UFH or LMWH including Heparin-Induced Thrombocytopenia (HIT), signs, or symptoms of venous thrombosis, severe renal and/or hepatic impairment, malignancy, high risk of bleeding and/or receiving warfarin. 

*Study groups*

The patients in one of the study groups received 40 mg of PDxane^®^ (Pooyesh Darou Biopharmaceuticals company, Tehran, Iran, prefilled syringes) by subcutaneous injection daily. The patients in another group received 40 mg of Clexane^® ^(Sanofi Aventis, Guildford, United Kingdom, prefilled syringes) by subcutaneous injection daily. The first dose of the medication was administered in the hospital under supervision of a physician following surgery procedure when the stability of the patients’ homeostasis was ascertained. Following discharge, the patients self-administered the medication at home for 10 days. This study was an open label research and the researchers provided the medication for the patients. The patients were allocated to two groups using block randomization method.

*Study outcomes and data collection*

The primary outcome of this study was prevention of VTE and was evaluated by signs and symptoms of thrombosis. In case of a suspected VTE event, further diagnostic procedures were considered. In cases where distal VTE was suspected, defined as a simplified Wells score of ≥ 2, ultrasonography of the suspected limb was ordered in order to confirm the diagnosis (23). 

In cases of the suspected proximal VTE in low risk patients with simplified Wells score of 0-1 and no clinical deterioration, D. Dimer levels were assessed. In the patients with D. Dimer levels more than age × 10 mcg/liter, Computed Tomography (CT) angiography was ordered. In high risk patients immediate CT angiography was conducted (24). 

Secondary outcomes of the study were frequency and severity of different adverse drug reactions observed with each preparation of medication. The patients were given clear instructions to report any adverse effects or signs of VTE immediately. At the end of VTE prophylaxis period, the patients were evaluated in the clinic. Data regarding relevant clinical and laboratory examinations including development of anemia defined as hemoglobin concentration less than 8 g/dL, changes in platelet counts, abnormal liver function test, rates of hematoma or hemorrhage, anaphylactic reactions, nausea, vomiting, central nervous system adverse reactions and cutaneous reactions in injections site were recorded. Other characteristics of the study population, including patient’s height, weight, comorbidities, gender, and habitual histories were also recorded.

*Statistical analysis*

Statistical analysis was carried out using SPPS software version 23. Mean (SD) and frequency (%) of the data were used for study population description. *P *< 0.05 was considered significant. Independent sample *t*-test method was considered to compare the mean and mean change of continuous outcomes between two groups. In order to analyze qualitative data, Pearson’s Chi-square test was applied. 

## Results

**Figure 1:**
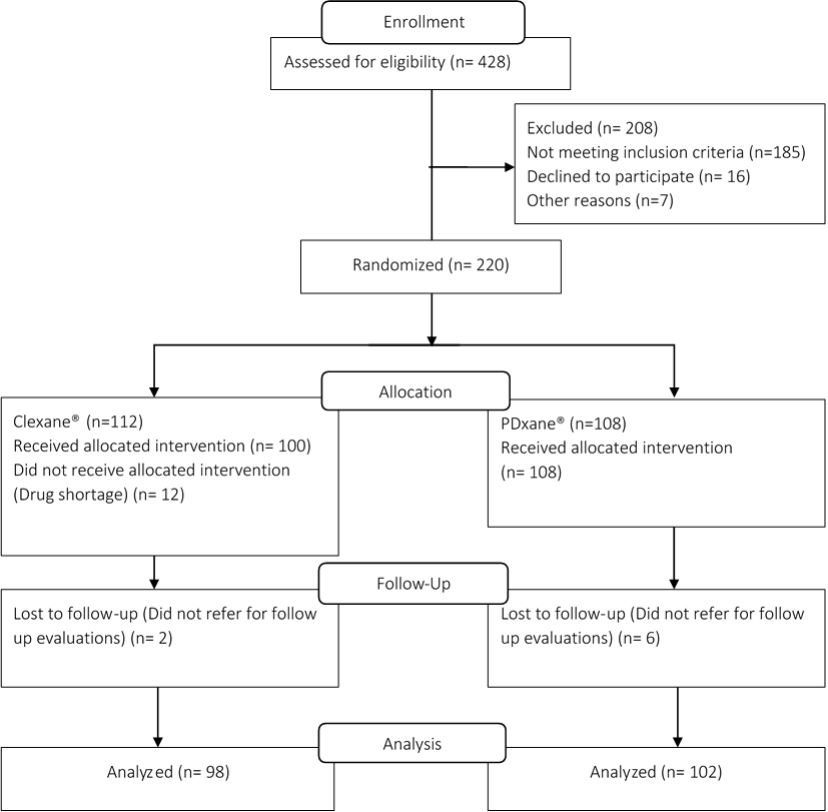
Flow diagram of the study participants

Among 220 patients who were enrolled in the study, 200 patients remained for final analysis. In the Clexane^®^ group 12 patients did not receive the allocated intervention due to noncompliance with the given instructions. Moreover, 2 and 6 patients did not return to the clinic for final evaluation in Clexane^®^ and PDxane^®^ groups, respectively. Figure 1 illustrates the flow diagram of the study participants. Demographic and baseline clinical characteristics of the patients are displayed in Table 1. As it could be noted, there was no significant difference between two groups of the patients considering baseline characteristics. None of the patients included in this study were immobile for the prolonged durations, and no patient with cancer was included in this study. The main risk factor that was present in the study population was obesity and its prevalence was not different between the study groups (9).

No cases of proximal or distal DVT or PE were observed in the study groups. The laboratory examination results of the patients in two study groups are summarized in Table 2. One patient (0.98%) in the PDxane^®^ group developed anemia, compared to none in the Clexane^®^ group but the difference was not significant (*P = *0.326). Two patients (1.96%) in PDxane^®^ group showed a rise in Aspartate transaminase (AST) levels to 3 times the upper limit of normal while no patient in Clexane^®^ group developed AST rise (*P = *0.164). 

Alanine transaminase (ALT) levels of four patients (3.92%) in PDxane^®^ group and three patients (3.06%) in Clexane^® ^group rose to more than 3 times of upper limit of normal, with no statistically significant difference (*P = *0.661).

No cases of significant or life threatening bleeding were observed in this study with either of the brands. 

The frequencies of observed enoxaparin-related adverse effects in two groups of the patients are summarized in Table 3. There was no significant difference in the development of injection site reactions, confusion, and hematuria between two groups of the patients.

**Table 1:** Demographic and base line characteristics of patients

		**PDxane® (N = 102)**	**Clexane® (N = 98)**	***P***
Age (Year), Mean (SD)		38.4 (10.40)	37.2 (10.25)	0.428
Weight (kg), Mean (SD)		73.5 (12.33)	74.4 (11.92)	0.573
Body Mass Index (kg/m2), Mean (SD)		28.1 (10.51)	28.6 (7.64)	0.641
Female Sex, N (%)		102 (100%)	98 (100%)	
Medical History	Diabetes mellitus, N (%) Hypertension, N (%)	6 (5.88) 8 (7.8)	6 (6.12) 10 (10.2)	0.943 0.560
	Cesarean	59 (57.8)	56 (57.1)	
Surgery type, N (%)	Total Abdominal Hysterectomy	37 (36.2)	41 (41.8)	0.151
	Other*	6 (5.8)	1 (1.02)	

*Total vaginal hysterectomy, Cesarean hysterectomy with or without colporrhaphy, Total abdominal hysterectomy with bilateral salpingo-oophorectomy.

**Table 2:** Results of laboratory examinations in two study groups.

	**PDxane® (N = 102)**	**Clexane® (N = 98)**	***P***
Before	11.6 (1.67)	11.8 (1.85)	
Hemoglobin (g/dL), Mean (SD)			0.326
After	12.26 (1.40)	12.6 (1.22)	
Before	221 (93.10)	227 (78.94)	
Platelet count (10^3), Mean (SD)			NA
After	327 (116.17)	341 (111.58)	
Before	25.64 (12.28)	22.4 (9.21)	
AST† (IU/mL), Mean (SD)			0.164
After	24.9 (12.58)	22.89 (9.98)	
Before	22.65 (17.32)	18.5 (13.18)	
ALT‡ (IU/mL), Mean (SD)			0.661
After	30.9 (39.05)	22.89 (17.85)	

†AST, Aspartate transaminase; ‡ALT, Alanine transaminase.

**Table 3:** Frequency (%) of enoxaparin-related adverse effects in two study groups

		**PDxane® (N = 102)**	**Clexane® (N = 98)**	***P***
Confusion		0	1 (1.02%)	0.306
Hematuria		2 (1.96%)	0	0.164
	Rash	1 (0.98%)	1 (1.02%)	0.977
Injection site reactions	Pain	6 (5.88%)	4 (4.08%)	0.559
	Erythema	6 (5.88%)	3 (3.06%)	0.336

## Discussion

Enoxaparin is a favored LMWH used frequently for VTE prophylaxis in patients (13, 14). With respect to high cost of its approved international branded preparations, providing a less expensive alternate product would be worthwhile. Comparative efficacy and safety would be specifically important in order to ascertain clinical similarity between a newly produced and previously approved preparation of enoxaparin. PDxane® showed similar efficacy and safety profile with Clexane® when used as VTE prophylaxis in this clinical trial of the patients underwent major obstetric-gynecological surgeries. PDxane® is about half the price of Sanofi Aventis product. Therefore, its use in clinical practice could lower the costs significantly. 

Few studies have compared the efficacy of different preparations of enoxaparin. In a study that was conducted in the patients underwent major abdominal surgeries, the clinical efficacy of Sanofi Aventis product was compared with a generic product by Eurofarma when used as VTE prophylaxis. The patients received the medications in a fixed dose of 40 mg subcutaneously for 7 to 10 days. The study results showed no significant difference in terms of efficacy between two preparations (25). This study had nearly large sample size and was conducted using appropriate imaging and laboratory techniques to assess the outcomes accurately. 

However, no data regarding types of the surgeries was reported in the paper. This is while surgery type could affect the risk of VTE and should be considered in interpretation of the results. 

In another study conducted by Casella IB *et al*. the efficacy of a generic enoxaparin preparation was compared with Sanofi Aventis product for treatment and prevention of VTE in 114 patients that underwent vascular surgeries. In terms of anti Xa activity and clinical efficacy, no significant difference was observed between two preparations (26). However, the number of patients in this study should be considered as a major limitation when its results are interpreted.

We observed similar safety profile from PDxane^®^ and Clexane^®^ in our study patients. According to our results, non of the preparations of enoxaparin resulted in serious adverse effects such as major bleeding or thrombocytopenia when used with prophylactic fixed doses of 40 mg SC. The most frequent adverse effect observed in our study was injection site reaction. This ADR could be prevented considering appropriate patient education and training regarding administration instructions. Frequency of this ADR was not significantly different between two groups confirming comparable safety of PDxane^®^. 

Other adverse effects that were observed in the current study included liver function test abnormalities and anemia. The rates of these adverse effects were not significantly different between two groups. Such adverse reactions have also been also reported in previous studies with various preparations of enoxaparin (27). No case of overt hepatitis was observed in both study groups and the liver function tests returned to normal following enoxaparin discontinuation. Two cases of hematuria that were observed in PDxane^®^ group relieved the following drug discontinuation. 

Our clinical trial suffered from some limitations. Comparing the efficacy and safety of two preparations of enoxaparin when used only for VTE prophylaxis in a specific group of the patients could affect the comprehensiveness of the study results. On the other hand, follow up period of 10 days following patient discharge might not be long enough to observe VTE episodes in the patients with prolonged risk factors for VTE (9, 10). Near one-third (38%) of our study patients were obese (BMI > 29 kg/m^2^) and obesity has been suggested as a major persistent risk factor for VTE (28). Therefore, longer follow-up duration might have been needed to compare the efficacy of enoxaparin for VTE prophylaxis in these patients. Further studies would be required in order to ascertain the clinical comparability of the studied preparations of enoxaparin for other indications and in other settings. 

In according to the study results PDxane^®^ was of similar efficacy and safety compared to Clexane^®^ in preventing VTE events during 10 days following major obstetric-gynecological surgeries. Considering lower cost of PDxane^®^, it could be a safe and effective alternate for VTE prophylaxis in the patients undergoing major obstetric-gynecological surgeries. 
